# Alpha-lipoic acid ameliorates nab-paclitaxel-induced peripheral neuropathy by inhibiting IL-17 signaling pathway

**DOI:** 10.3389/fimmu.2025.1674709

**Published:** 2025-11-28

**Authors:** Hong Sun, Yuxin Cai, Ling Li, Xiu Xin, Jingchao Yan, Taomin Huang

**Affiliations:** 1 Department of Pharmacy, Eye & ENT Hospital, Fudan University, Shanghai, China; 2 Department of Clinical Pharmacy and Regulatory Science, School of Pharmacy, Fudan University, Shanghai, China

**Keywords:** alpha-lipoic acid, nab-paclitaxel, peripheral neuropathy, network toxicology, network pharmacology

## Abstract

**Background:**

The precise mechanisms by which alpha-lipoic acid (LA) alleviates nab-paclitaxel (nab-PTX)-induced peripheral neuropathy have yet to be fully elucidated. The objective of this study was to investigate the mechanisms underlying the neuroprotective effects of LA in mitigating nab-PTX-induced peripheral neuropathy.

**Methods:**

We established a rat model of nab-PTX-induced peripheral neuropathy to evaluate the efficacy of LA. To systematically elucidate the mechanisms by which LA alleviates nab-PTX-induced peripheral neuropathy, we utilized an integrated approach that combined network toxicology and network pharmacology. Subsequently, molecular docking analysis was performed to assess the binding affinity of the LA to the target proteins involved in the key signaling pathway. Furthermore, experimental validation was conducted to confirm the role of the key signaling pathway in the neuroprotective mechanism of LA.

**Results:**

LA was demonstrated to effectively alleviate nab-PTX-induced peripheral neuropathy. The network analysis indicated that LA ameliorated nab-PTX-induced peripheral neuropathy primarily through the AGE-RAGE signaling pathway in diabetic complications, IL-17 signaling pathway, fluid shear stress and atherosclerosis, NOD-like receptor signaling pathway, and pathways of neurodegeneration - multiple diseases. The molecular docking indicated a potential impact of LA on the IL-17 signaling pathway. Further experiment validation revealed that nab-PTX activated the IL-17 signaling pathway, whereas LA could mitigate nab-PTX-induced peripheral neuropathy by inhibiting this pathway.

**Conclusion:**

By integrating network toxicology analysis, network pharmacology analysis, and experimental validation, this study provides a clearer understanding of the mechanisms by which LA ameliorates nab-PTX-induced peripheral neuropathy.

## 1 Introduction

Nanoparticle albumin-bound paclitaxel (nab-PTX) is a widely used chemotherapeutic agent that has demonstrated efficacy in various cancers (1–6). However, it induces a high incidence and severity of peripheral neuropathy, which represents a major dose-limiting toxicity that severely compromises patients’ quality of life and treatment outcomes (7–9). The increased neurotoxicity of nab-PTX, compared to solvent-based paclitaxel, is likely attributable to its improved delivery and higher accumulation in peripheral nerves (10, 11). Unfortunately, efficient therapeutic strategies to manage this complication are largely limited. Therefore, it is essential to monitor peripheral neuropathy during nab-PTX treatment, and there is an urgent need to identify effective strategies to mitigate its development.

Alpha-lipoic acid (LA), a natural antioxidant with established efficacy in mitigating diabetic peripheral neuropathy (DPN), has shown promise in alleviating chemotherapy-induced peripheral neuropathy (CIPN) (12–15). Its potential protective effects against nab-PTX-induced neuropathy, however, remain incompletely understood. While previous research on LA has illuminated LA’s impact on isolated pathways like Nrf2, the comprehensive network of molecular targets and the primary signaling pathways through which LA counteracts nab-PTX neurotoxicity are still elusive (16).

Given the multifactorial pathogenesis of peripheral neuropathy, a systematic approach is crucial for elucidating complex drug actions. In this study, we employed an integrative strategy combining network toxicology and network pharmacology to holistically predict the potential targets and pathways involved in LA’s protection against nab-PTX-induced peripheral neuropathy (17, 18). The integrated approach might help us identify multiple signaling pathways through which LA mitigates nab-PTX-induced peripheral neuropathy, such as Nrf2/ARE, NF-κB, PI3K/Akt and others. Experimental validation was then performed to confirm the role of the key signaling pathway in the neuroprotective mechanism. Our study addresses the limitations of previous research, which usually focused on isolated aspects, thereby providing a more comprehensive and systematic investigation into the mechanisms by which LA alleviates nab-PTX-induced peripheral neuropathy.

## 2 Materials and methods

### 2.1 Drugs and reagents

The lipoic acid injection (Yabao Pharmaceutical Group Co. Ltd.), nab-PTX injection (Jiangsu Hengrui Pharmaceuticals Co., Ltd), and normal saline injection (Sichuan Kelun Pharmaceutical Co., Ltd.a) were obtained from Eye Ear Nose Throat Hospital of Fudan University. The enzyme-linked immunosorbent assay (ELISA) kits for IL-17, TNF-α, and MMP-13 were obtained from Shanghai Jianglai Biotechnology Co., Ltd., while the kit for HSP-90α was sourced from UpingBio.

### 2.2 Animals and treatment

Twenty male Sprague-Dawley (SD) rats were procured from Shanghai Bikai Keyi Biotechnology Co., Ltd., and were housed under standardized laboratory conditions, which included a 12-hour light/dark cycle, ambient temperature of 25°C, and relative humidity maintained between 55% and 60%. The animals were free access to food and water throughout the study. The experimental procedures adhered strictly to the guidelines and recommendations set forth by the International Association for the Study of Pain. Every effort was made to minimize both the number of animals utilized and their potential suffering throughout the study. After a one-week adaptation period, the rats were randomly assigned into five groups (n=4 per group) using a computer-based random order generator: control group (administered normal saline), nab-PTX group (10 mg/kg), low dose LA (LLA)+nab-PTX group (nab-PTX 10 mg/kg combined with 15 mg/kg LA), medium dose LA (MLA)+nab-PTX group (nab-PTX 10 mg/kg combined with 30 mg/kg LA), and high dose (HLA)+nab-PTX group (nab-PTX 10 mg/kg combined with 60 mg/kg LA). The nab-PTX was administered once a week for three consecutive weeks via tail vein injection; LA was given once daily via intraperitoneal injection. The assessment of peripheral neuropathy was conducted in a blinded manner with respect to drug administration on days 0, 1, 8, 15, and 22. This experiment was approved by the Experimental Animal Ethics Committee of School of Pharmacy Fudan University (2025-03-LY-GY-44). Rats were excluded from the study if they died prematurely, as this would prevent the collection of complete behavioral and histological data.

### 2.3 Peripheral neuropathy assessment

We employed the fixed-threshold von Frey method to assess the mechanical pain of rats (19). Each rat was individually placed in a transparent chamber (20×10×20 cm) containing a specially designed iron wire mesh platform, which formed a uniform 10 mm grid. Seven calibrated von Frey filaments (1, 2, 4, 6, 8, 10, and 15 g) were applied in ascending order to the central area of the plantar surface of one hind paw. Each filament was pressed against the midplantar skin five times until it slightly bent, and then held in place for 5 seconds. Trials consisted of five applications of each filament to the hind paw, with a 15-second interval between each application. If the hind paw withdrew in response to a particular filament in at least 4 out of 5 applications, the force value of that filament (in grams) was defined as the paw withdrawal threshold (PWT). The withdrawal responses from both hind paws were documented, and the measurements from each paw were analyzed separately. The percentage response for the 4 g, 8 g, and 15 g von Frey filaments was calculated. Acetone test was performed to assess the cold allodynia of rats (20). A 0.05 ml drop of acetone was delicately applied to the central region of the ventral surface of the rat’s hindpaw using a 1-ml syringe. Utmost care was exercised to prevent any contact between the syringe tip and the hindpaw, thereby avoiding any mechanical stimulation that could confound the results. The rats’ responses to acetone application were assessed using a scoring system. A score of 0 indicated no cold allodynia, while 1, 2, and 3 corresponded to mild (scratching the hind paw), moderate (intense licking, biting, or withdrawal of the hind paw), and severe (vigorous licking, multiple withdrawals, or vocalizations) cold allodynia, respectively. Five trials were carried out at intervals of approximately 5 min for each hind paw. The observation window for each trial was 30 seconds, during which the rats were observed continuously, and the highest score observed within this period was recorded for each trial. The ambient temperature in the testing room was maintained at 25 ± 1 °C to ensure a stable and controlled environment for the experiments. The statistical analysis was performed using one-way ANOVA followed by Dunnett’s *post-hoc* test.

### 2.4 Network toxicology analysis

The potential mechanisms underlying nab-PTX-induced peripheral neuropathy were explored using network toxicology. The SwissTargetPrediction (http://www.swisstargetprediction.ch/) (21), DrugBank (https://go.drugbank.com/) (22), and Comparative Toxicogenomics Database (CTD) (https://ctdbase.org/) databases (23) were utilized to identify the potential targets of nab-PTX. Due to the relatively limited number of targets associated with nab-PTX, we retained all the potential targets. The pathological targets associated with peripheral neuropathy were obtained from the GeneCards (https://www.genecards.org/; relevance score > median value) and Online Mendelian Inheritance in Man (OMIM) (https://omim.org/) databases (24). Venn diagram was employed to identify the targets potentially involved in nab-PTX-induced peripheral neuropathy. The Protein-Protein Interaction (PPI) network was constructed using the STRING database (https://cn.string-db.org/), with interactions filtered based on a medium confidence score (25). Gene Ontology (GO) and Kyoto Encyclopedia of Genes and Genomes (KEGG) enrichment analyses were performed using the DAVID (https://davidbioinformatics.nih.gov/tools.jsp) (26). The visualization of enrichment analysis was plotted using the Chiplot platform (https://www.chiplot.online/) (27).

### 2.5 Network pharmacology analysis

Network pharmacology was employed to investigate the potential protective effects of LA against nab-PTX-induced peripheral neuropathy. The SwissTargetPrediction, DrugBank, and CTD were used to identify the potential targets of LA. We had also retained all the potential targets of LA. The overlapping targets between LA and peripheral neuropathy were determined using Venn diagrams. Additionally, PPI network analysis with interactions filtered based on a medium confidence score, GO enrichment analysis, and KEGG pathway analysis were conducted to further elucidate the mechanisms.

### 2.6 Integration analyses of network toxicology and network pharmacology

The potential mechanisms by which LA ameliorates nab-PTX-induced peripheral neuropathy were initially identified through network toxicology and network pharmacology approaches. To achieve a more precise understanding of these mechanisms, further integrated analyses combining network toxicology and network pharmacology were performed. The overlapping targets among nab-PTX, LA, and peripheral neuropathy were identified using Venn diagrams. Additionally, PPI network analysis and KEGG pathway analysis were conducted to provide deeper insights into the underlying mechanisms.

### 2.7 Molecular docking analysis

The three-dimensional structure of LA was obtained from the PubChem Compound database (https://www.ncbi.nlm.nih.gov/pccompound/). Meanwhile, the crystal structure of the protein was sourced from the Protein Data Bank (PDB) database (https://www.rcsb.org/). Following this, the files of receptor and ligand were processed via the CB-Dock2 portal to carry out molecular docking analysis (28).

### 2.8 Validation of the IL-17 signaling pathway

All rats were put under deep anesthesia with a ketamine after the trial was finished. The serum, spinal cords and dorsal root ganglia (DRG) were collected and kept in liquid nitrogen in a freezer at a temperature of -80°C. The levels of IL-17, HSP-90α, TNF-α, and MMP-13 in serum, spinal cords, and DRG were measured using enzyme-linked immunosorbent assay (ELISA) kits, following the manufacturer’s instructions. The statistical analysis was performed using one-way ANOVA followed by Dunnett’s *post-hoc* test.

## 3 Results

### 3.1 LA ameliorated nab-PTX-induced peripheral neuropathy

**Figure 1:**
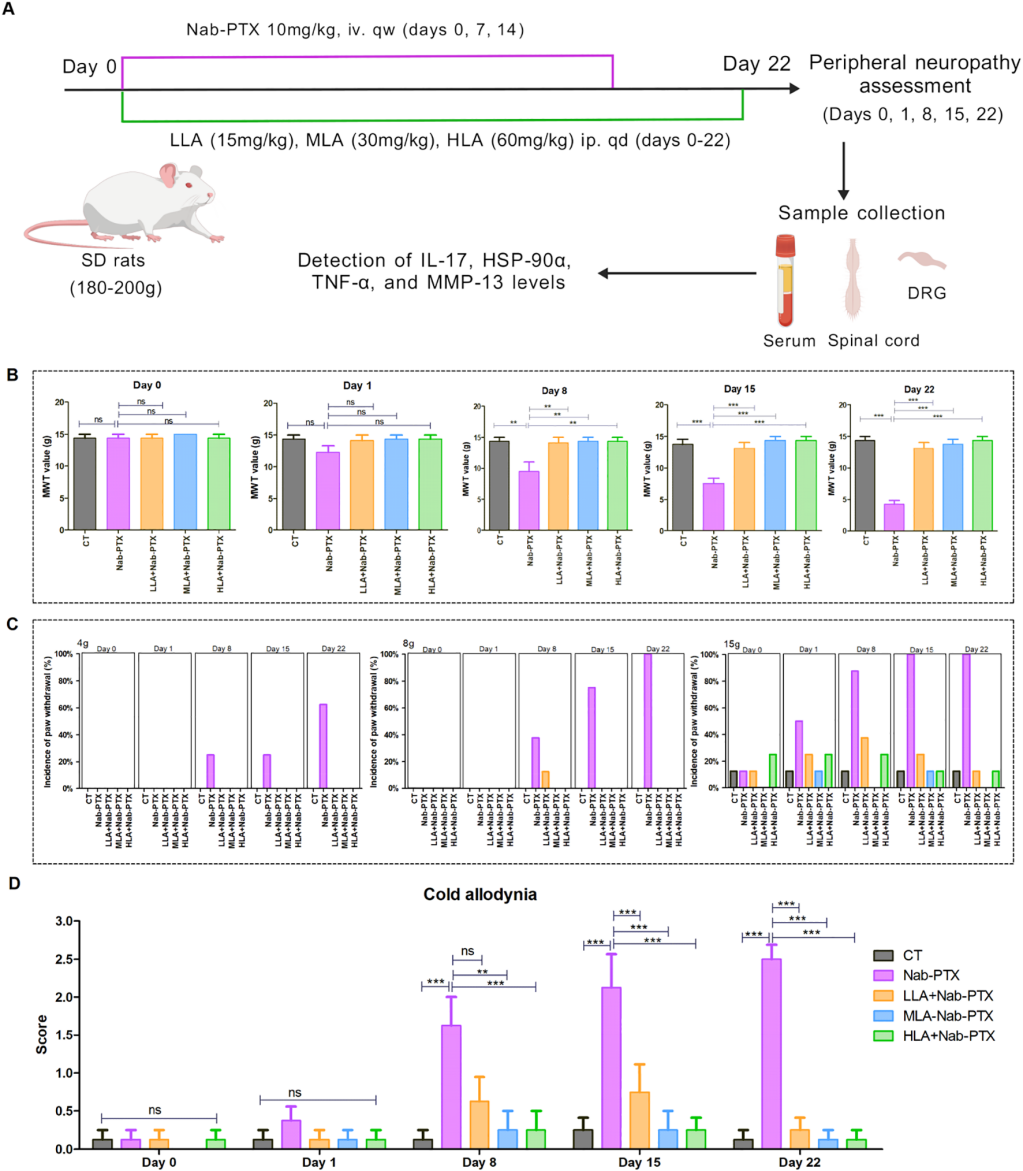
Neuroprotective effects of LA in rats of nab-PTX-induced peripheral neuropathy. **(A)** Schematic representation of animal experiment [created with biogdp.com (29)]; **(B, C)** Results of mechanical allodynia tests (n=8 paws); **(D)** Results of cold allodynia tests (n=8 paws). **P<0.01, ***P<0.001.

To investigate mechanical and cold allodynia, we performed von Frey and acetone tests on days 0, 1, 8,15, 22 after administration (**Figure 1A**). The results demonstrated that rats in the treatment of nab-PTX exhibited a significant decrease in bilateral paw withdrawal thresholds compared to those in the control group (**Figure 1B**). In terms of 4, 8, and 15 g von Frey tests, rats treated with nab-PTX exhibited significantly more responses than those in the control group (**Figure 1C**). Similarly, the acetone test for cold allodynia revealed that nab-PTX-treated rats had significantly higher scores compared to controls (**Figure 1D**). LA demonstrated significant effects on nab-PTX-induced neuropathic pain. Rats treated with LA (in three dosage regimens) in combination with nab-PTX showed a significant increase in mechanical withdrawal thresholds compared to those treated with nab-PTX alone (**Figure 1B**). Regarding 4, 8, and 15 g von Frey tests, rats treated with LA plus nab-PTX exhibited significantly fewer responses than those treated with nab-PTX alone (**Figure 1C**). Additionally, the cold allodynia scores of rats treated with LA plus nab-PTX were significantly lower compared to those treated with nab-PTX alone (**Figure 1D**). These results collectively suggested that LA could effectively alleviate mechanical and cold allodynia caused by nab-PTX. Rats treated with nab-PTX exhibited a slight decrease in body weight (**Supplementary Figure S1**).

### 3.2 Network toxicology analysis of the potential mechanisms of nab-PTX-induced peripheral neuropathy

**Figure 2:**
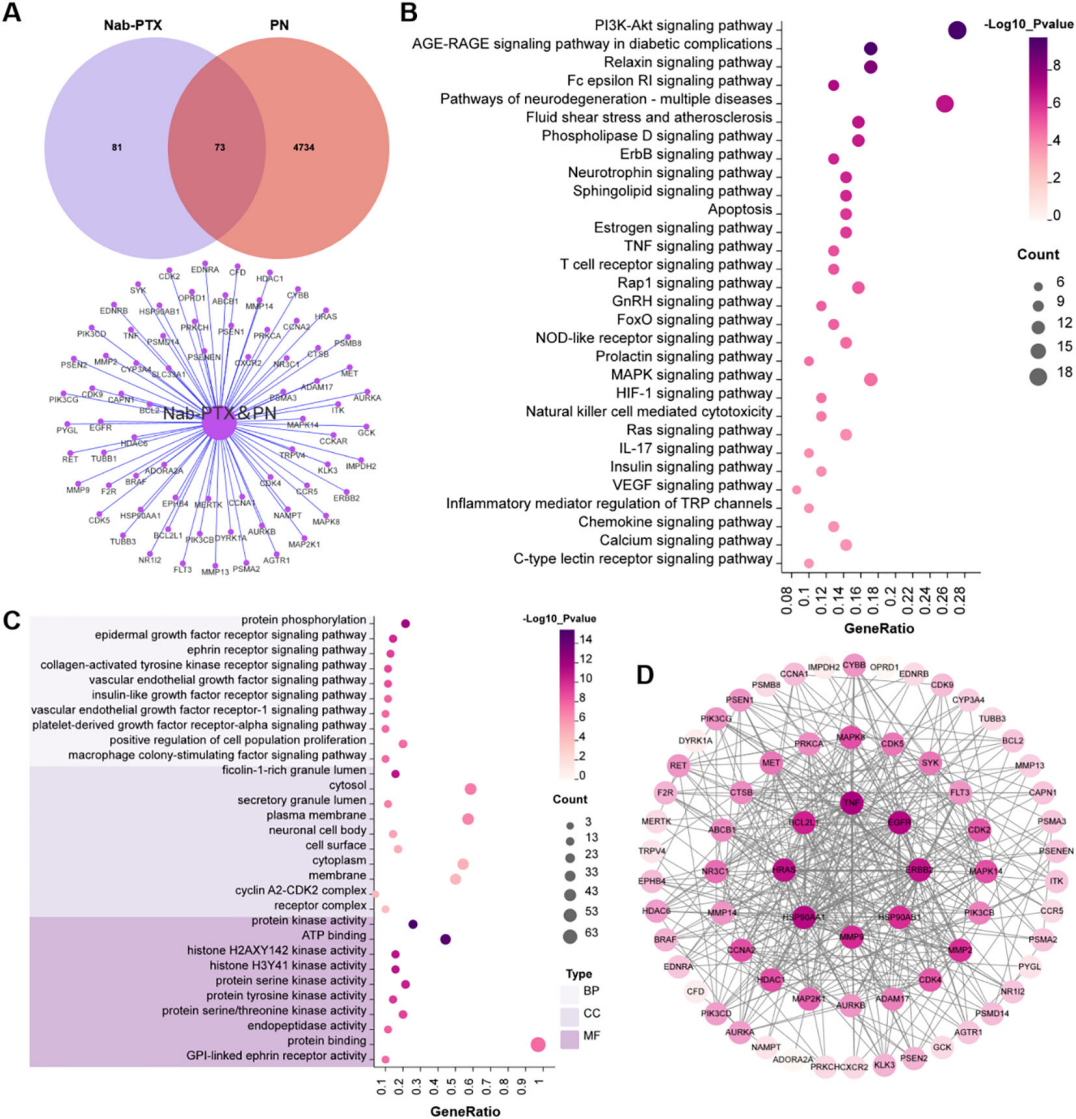
Results of network toxicology analysis. **(A)** Venn diagram; **(B)** KEGG analysis; **(C)** GO enrichment analysis; **(D)** PPI network.

A total of 154 target genes were identified in association with nab-PTX, while 4807 target genes were linked to peripheral neuropathy. Upon further analysis, an intersection of these two sets of genes identified 73 common target genes (**Figure 2A**). The PPI network of these targets was illustrated in **Figure 2D**. The network was constructed based on the intricate interactions among the core targets. This optimized network diagram provided a clear visual representation of the relationships and functional associations among these core targets. GO enrichment analysis showed that, for biological processes (BP), the genes were predominantly involved in protein phosphorylation, epidermal growth factor receptor signaling pathway, ephrin receptor signaling pathway, and collagen-activated tyrosine kinase receptor signaling pathway. It suggested that kinase-mediated signal transduction disorder could be the core mechanism behind nerve damage. Regarding cellular components (CC), the genes were mainly localized to the ficolin-1-rich granule lumen, cytosol, secretory granule lumen, and plasma membrane, hinting abnormal secretion of neurotransmitters/inflammatory factors and dysfunction of membrane receptors may be involved in the toxic process. In terms of molecular functions (MF), the genes were primarily enriched in protein kinase activity, ATP binding, histone H2AXY142 kinase activity, and histone H3Y41 kinase activity (**Figure 2C**), which further confirmed the driving effect of kinase activity imbalance in neuropathy. KEGG enrichment analysis highlighted the involvement of these genes in key pathways such as PI3K-Akt signaling pathway, AGE-RAGE signaling pathway in diabetic complications, fluid shear stress and atherosclerosis pathway, TNF signaling pathway, and IL-17 signaling pathway (**Figure 2B**). The result of KEGG enrichment analysis denoted that inflammatory response, oxidative stress, and imbalanced neurotrophic support are the core pathological basis of nab-PTX induced peripheral neuropathy.

### 3.3 Network pharmacology analysis of the potential neuroprotective mechanism of LA

**Figure 3:**
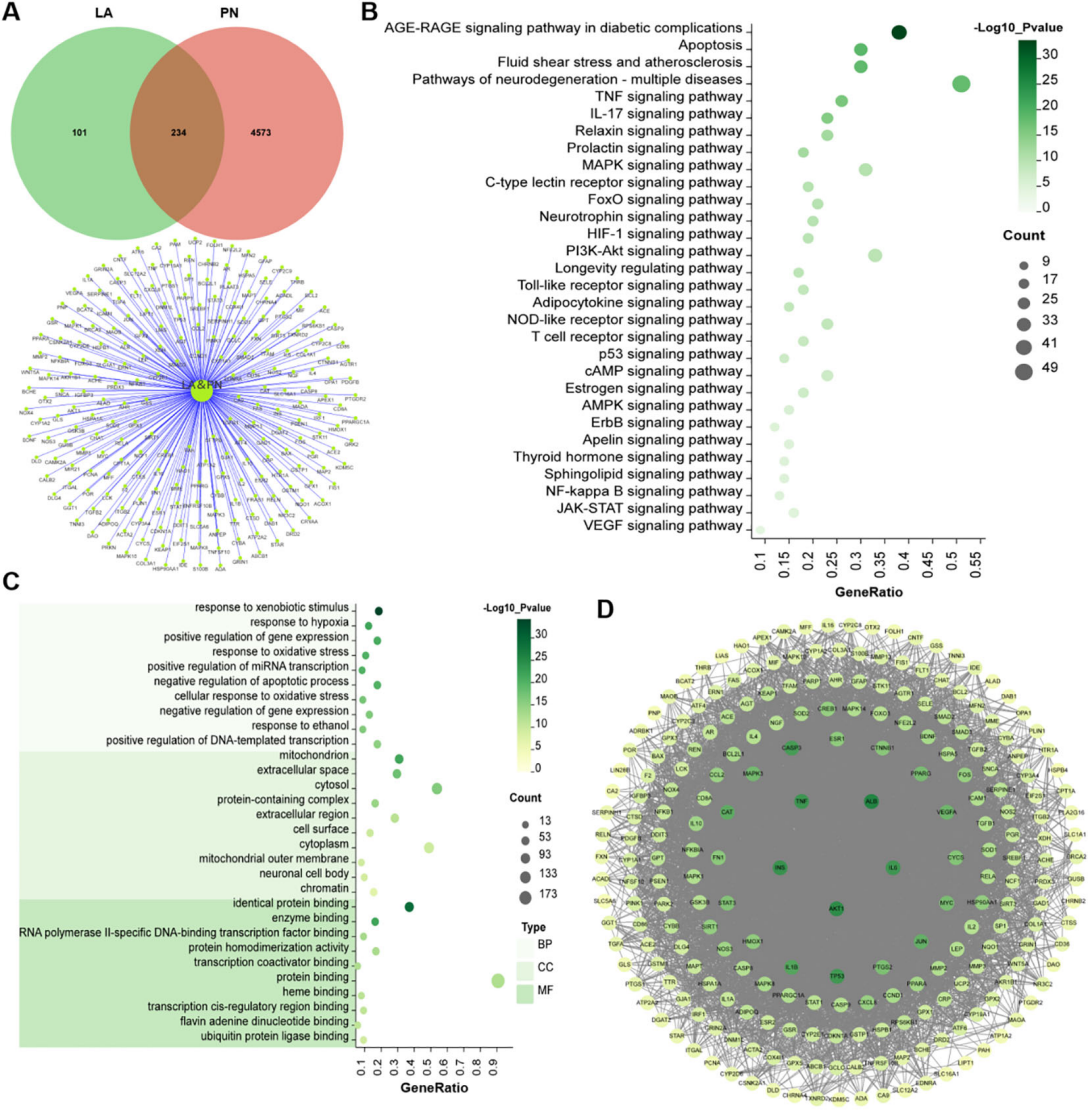
Results of network pharmacology analysis. **(A)** Venn diagram; **(B)** KEGG analysis; **(C)** GO enrichment analysis; **(D)** PPI network.

As shown in **Figure 3A**, a total of 335 genes were identified in association with LA. Among these, 234 genes were found to overlap with those implicated in peripheral neuropathy. The PPI network exhibited a highly complex structure (**Figure 3D**). The GO enrichment analysis revealed that LA primarily influenced biological processes associated with response to xenobiotic stimulus, response to hypoxia, positive regulation of gene expression, and response to oxidative stress (**Figure 3C**). It implied that LA had multidimensional protective effects such as antioxidant stress and anti-inflammatory effects. Additionally, KEGG enrichment analysis highlighted that the neuroprotective effects of LA were predominantly mediated through key pathways, including the AGE-RAGE signaling pathway in diabetic complications, apoptosis, fluid shear stress and atherosclerosis pathway, TNF signaling pathway, and IL-17 signaling pathway (**Figure 3B**).

### 3.4 Potential mechanism of LA ameliorating nab-PTX-induced peripheral neuropathy

**Figure 4:**
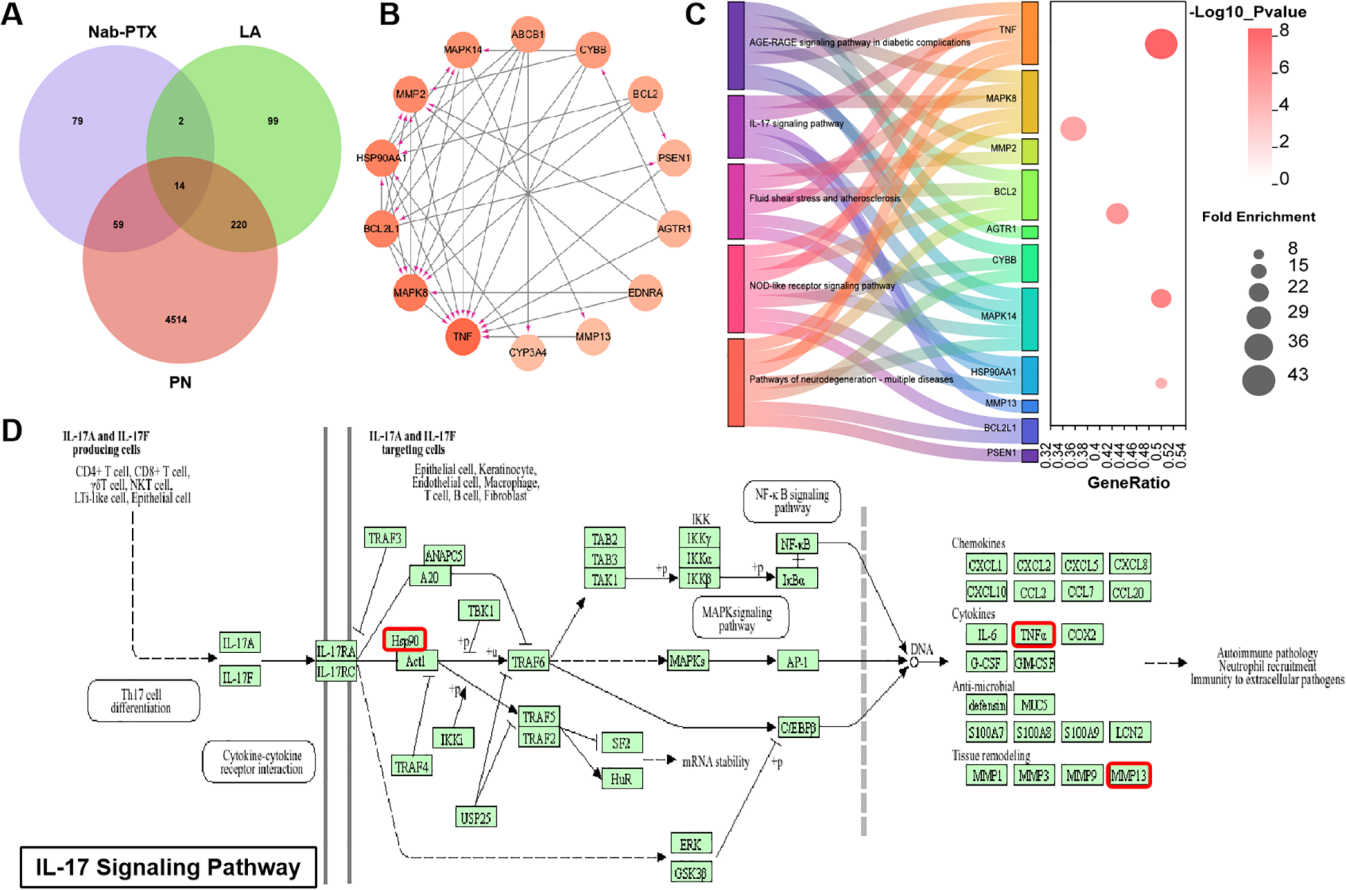
Results of the integrated analysis in network toxicology and network pharmacology. **(A)** Venn diagram; **(B)** PPI network; **(C)** KEGG analysis; **(D)** IL-17 signaling pathway (Pathway information generated by KEGG).

Based on the results of network toxicology and network pharmacology, we found that the mechanism by which LA ameliorates peripheral neuropathy induced by nab-PTX might be associated with multiple signaling pathways such as PI3K-Akt signaling pathway, AGE-RAGE signaling pathway in diabetic complications, TNF signaling pathway and the IL-17 signaling pathway. Further integrated analyses, combining approaches from network toxicology and network pharmacology, identified 14 common genes shared among nab-PTX, LA, and peripheral neuropathy (**Figures 4A, B**). KEGG analysis revealed that the key pathways involved in these common genes included the AGE-RAGE signaling pathway in diabetic complications, IL-17 signaling pathway (**Figure 4D**), fluid shear stress and atherosclerosis, NOD-like receptor signaling pathway, and pathways of neurodegeneration - multiple diseases (**Figure 4C**).

### 3.5 LA may ameliorate nab-PTX-induced peripheral neuropathy by IL-17 signaling pathway

**Figure 5:**
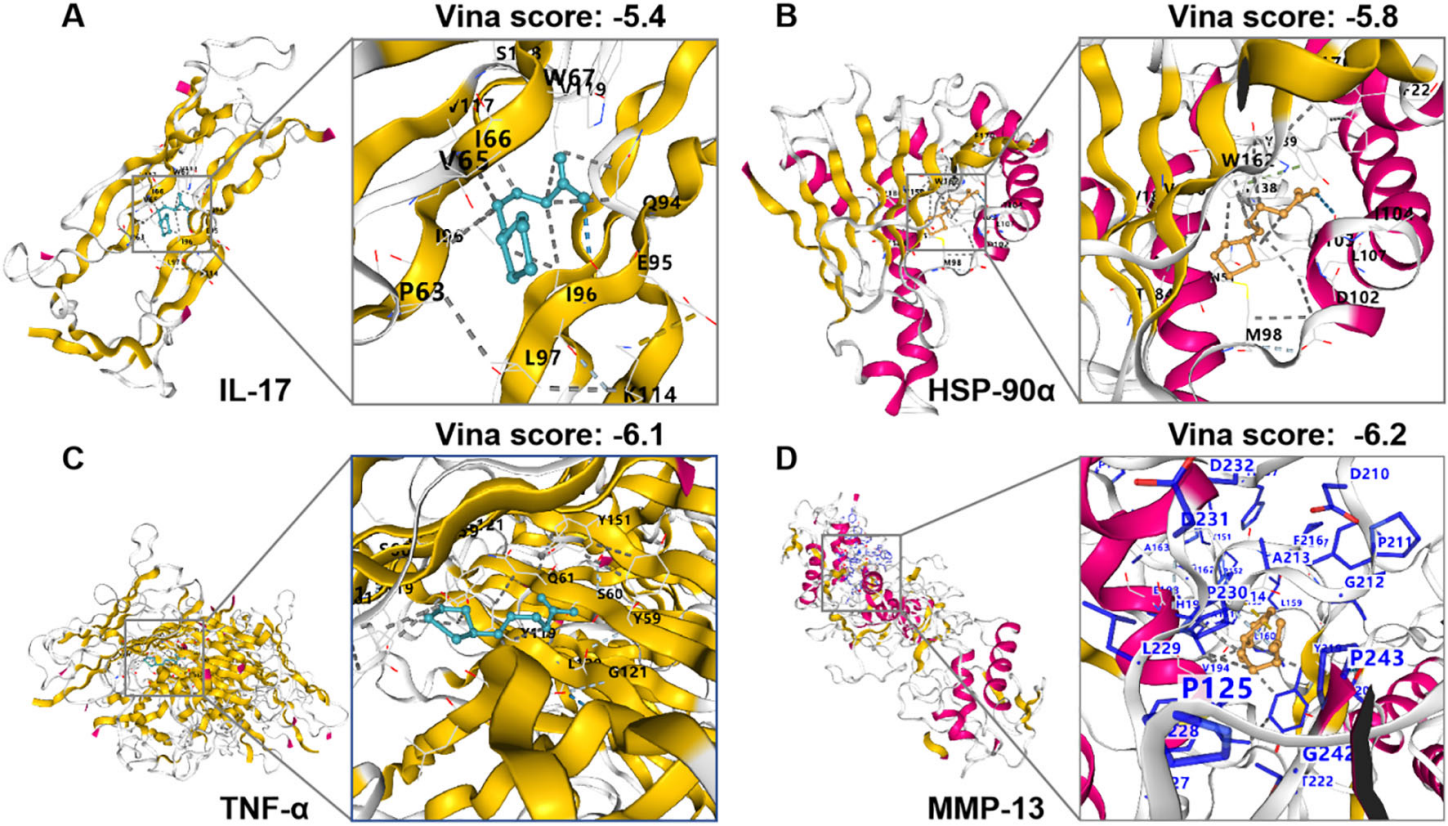
Docking results of LA with the **(A)** IL-17; **(B)** HSP-90α; **(C)** TNF-α; **(D)** MMP-13.

Based on the predictions of network toxicology and network pharmacology, we identified several potential pathways but prioritized the IL-17 signaling pathway for further validation. This is due to its well-documented role in neuroinflammation and allodynia, and its high concordance with the anti-inflammatory mechanisms of LA. The molecular docking analysis was performed to assess the binding affinity of the LA to the target proteins involved in IL-17 signaling pathway (**Figures 5A-D**). The PubChem CID of LA and the PDB IDs of all proteins employed for docking were provided in **Supplementary Table S1**. Given that all the results scored lower than −5.0 according to the Vina scoring system, this indicated that the ligand exhibited favorable binding interactions with the receptor. The findings suggest that LA may have a potential impact on the IL-17 signaling pathway.

### 3.6 LA ameliorated nab-PTX-induced peripheral neuropathy by inhibiting IL-17 signaling pathway

**Figure 6:**
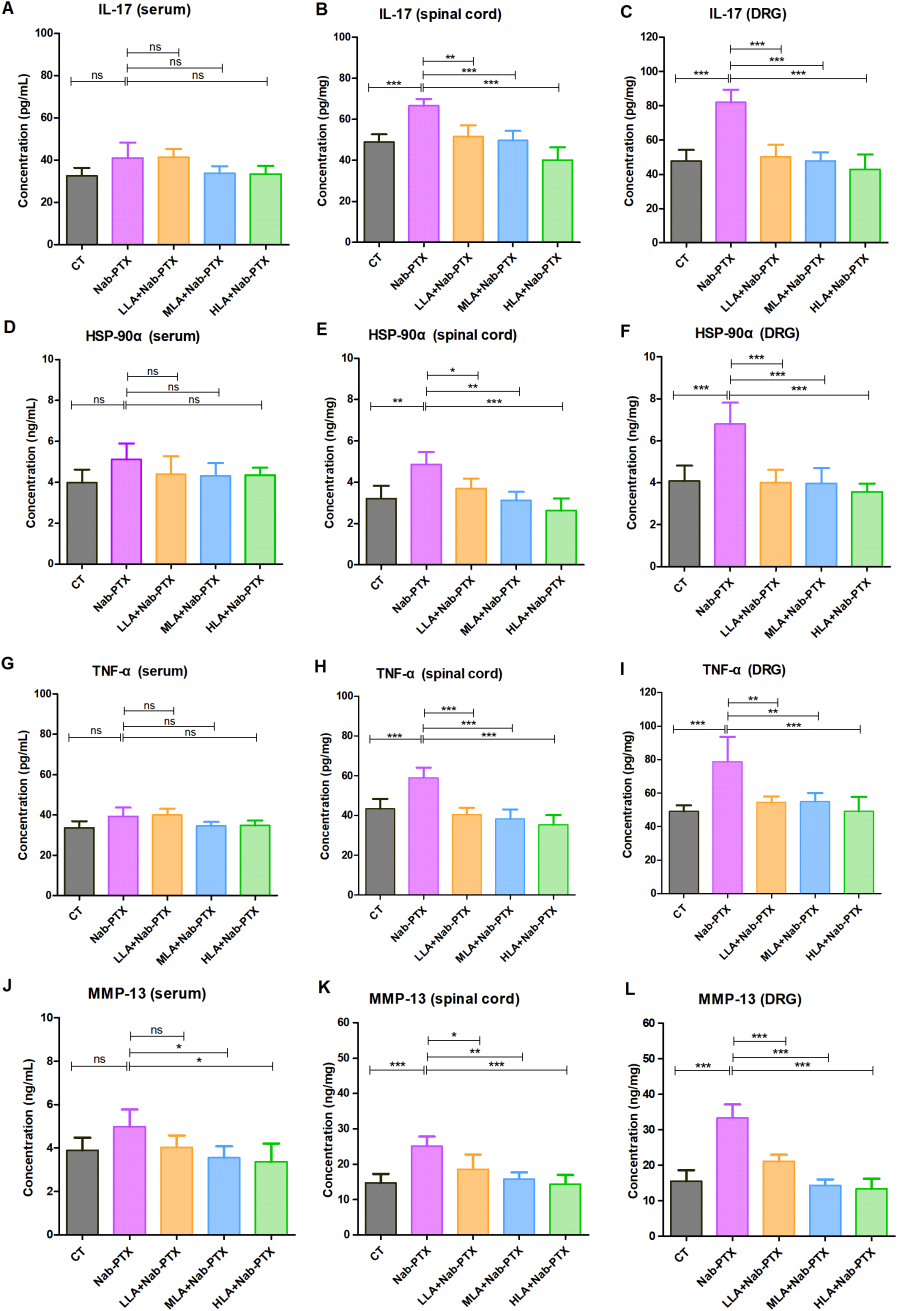
The detection results of IL-17, HSP-90α, TNF-α, and MMP-13. **(A)** IL-17 levels in serum; **(B)** IL-17 levels in spinal cord; **(C)** IL-17 levels in DRG; **(D)** HSP-90α levels in serum; **(E)** HSP-90α levels in spinal cord; **(F)** HSP-90α levels in DRG; **(G)** TNF-α levels in serum; **(H)** TNF-α levels in spinal cord; **(I)** TNF-α levels in DRG; **(J)** MMP-13 levels in serum; **(K)** MMP-13 levels in spinal cord; **(L)** MMP-13 levels in DRG. n=4, *P<0.05, **P<0.01, ***P<0.001.

IL-17 signaling pathway was selected for experimental validation. The levels of IL-17, HSP-90α, TNF-α, and MMP-13 in serum, spinal cord, and DRG of rats were presented in **Figure 6**. IL-17 expression analysis revealed distinct compartment-specific regulation. While serum IL-17 levels remained unchanged across all groups (**Figure 6A**), neural tissues showed significant pathway activation. In the spinal cord, nab-PTX treatment induced a substantial increase in IL-17 compared to controls (*P* < 0.05, **Figure 6B**), which was effectively normalized by LA co-treatment. A parallel pattern was observed in DRG tissues (**Figure 6C**), where nab-PTX elevated IL-17 levels and LA administration significantly suppressed this increase.

HSP-90α measurements demonstrated a similar tissue-specific profile. No significant inter-group differences were detected in serum HSP-90α (**Figure 6D**). However, both spinal cord (**Figure 6E**) and DRG (**Figure 6F**) tissues from nab-PTX-treated rats exhibited marked HSP-90α upregulation (*P* < 0.05), which was significantly attenuated by LA treatment.

TNF-α expression patterns followed the established trend. Serum TNF-α levels showed no treatment-induced alterations (**Figure 6G**). In contrast, spinal cord (**Figure 6H**) and DRG (**Figure 6I**) tissues from the nab-PTX group displayed significantly elevated TNF-α (*P* < 0.05), with LA treatment effectively reducing these levels toward baseline.

MMP-13 quantification revealed distinct regulatory patterns across compartments. Serum analysis showed no significant difference in MMP-13 levels between the control and nab-PTX groups (**Figure 6J**). In contrast, MLA and HLA treatment significantly reduced serum MMP-13 compared to nab-PTX group (*P* < 0.05). Meanwhile, in neural tissues, nab-PTX-induced MMP-13 upregulation was observed in both spinal cord (**Figure 6K**) and DRG (**Figure 6L**), with LA treatment significantly suppressing these increases (*P* < 0.05).

In summary, by targeting the IL-17 inflammatory axis in spinal cord and DRG, LA coordinately suppresses the expression of TNF-α, HSP-90α, and MMP-13, thereby blocking the neuroinflammation-injury cascade. Moreover, these findings indicate that even low-dose LA is sufficient to effectively curb the overactivation of the IL-17 pathway. These results not only validated prior network pharmacology predictions but provided mechanistically defined experimental evidence for LA’s therapeutic potential against CIPN.

## 4 Discussion

Peripheral neuropathy is a major dose-limiting side effect of nab-PTX, significantly impacting patient quality of life (30). The management of peripheral neuropathy induced by nab-PTX continues to be an important challenge for both clinicians and cancer patients. In clinical practice, if a patient develops peripheral neuropathy while being treated with nab-PTX, the clinicians usually reduce the dose or discontinue the use of nab-PTX for the patient. However, dose reduction or discontinuation of nab-PTX may compromise the efficacy of cancer treatment, highlighting the urgent need for effective strategies to manage and mitigate peripheral neuropathy without sacrificing therapeutic outcomes.

The pathogenesis of CIPN and DPN exhibits considerable overlap. Both conditions are underpinned by comparable pathophysiological processes, such as oxidative stress, neuroinflammation, mitochondrial dysfunction, and changes in neuronal ion channel activities (31). Therefore, theoretically, drugs that could alleviate DPN should also be effective for CIPN. LA is a versatile compound boasting a wide range of potential health benefits, including potent antioxidant, anti-inflammatory, and neuroprotective properties (32, 33). In our study, to elucidate the mechanisms underlying the neuroprotective effects of LA in mitigating nab-PTX-induced peripheral neuropathy, we initially conducted network toxicology and network pharmacology analyses. The former aimed to uncover the potential mechanisms of nab-PTX-induced peripheral neuropathy, while the latter focused on exploring how LA exerts its protective effects against peripheral neuropathy. Subsequently, we performed integrated analyses that combined insights from both network toxicology and network pharmacology to pinpoint the precise neuroprotective mechanisms of LA against nab-PTX-induced peripheral neuropathy. Finally, we selected the IL-17 signaling pathway for validation through molecular docking analysis and experimental approaches. Through a comprehensive approach combining network toxicology analysis, network pharmacology analysis, molecular docking analysis, and experimental validation, we have systematically elucidated the molecular mechanisms underlying LA’s therapeutic effects against nab-PTX-induced peripheral neuropathy. Our network analysis has unveiled that the mechanism underlying the amelioration of nab-PTX-induced peripheral neuropathy by LA might be intricately linked to multiple signaling pathways. These pathways mainly include the AGE-RAGE signaling pathway in diabetic complications, apoptosis, fluid shear stress and atherosclerosis pathway, TNF signaling pathway, and IL-17 signaling pathway. Molecular docking analysis indicates a potential impact of LA on the IL-17 signaling pathway. Further experimental validation has elucidated that nab-PTX could activate the IL-17 signaling pathway. In contrast, LA could attenuate nab-PTX-induced peripheral neuropathy by inhibiting this pathway. The novelty of our study lies in the systematic identification and subsequent validation of the IL-17 signaling pathway as an important mechanism for LA’s effect specifically in the context of nab-PTX-induced peripheral neuropathy, which to our knowledge, has not been reported before. Previous studies mainly focused on LA’s antioxidant properties, our integrated approach of network toxicology/pharmacology and experimental validation provides a new mechanistic insight into its immunomodulatory role.

IL-17 is a pro-inflammatory cytokine that plays a crucial role in modulating immune responses, neuroinflammation and is implicated in pain regulation (34, 35). IL-17 was reported to play roles in neuropathic pain and neurological disorders (36–38). Research has shown that IL-17 may exacerbate neuropathic pain by stimulating the proliferation of astrocytes and increasing the secretion of pro-inflammatory cytokines in models of spinal nerve ligation-induced neuropathic pain (39). TNF-α is a cytokine primarily produced by immune cells, and it plays a significant role in immune responses, inflammatory processes, and apoptosis. TNF-α also plays a pivotal role in the pathogenesis of peripheral neuropathic pain (40). HSP-90α is a multifunctional protein that plays critical roles in maintaining cellular homeostasis, responding to stress, and regulating key biological processes (41). MMP-13, a member of the peptidase M10 family of matrix metalloproteinases (MMPs), has been reported to promote paclitaxel-induced peripheral neuropathy (42, 43). These targets were involved in IL-17 signaling pathway.

Our findings demonstrated that in nab-PTX-induced peripheral neuropathy, IL-17 pathway activation occurs predominantly within neural microenvironments (spinal cord/DRG). By targeting the IL-17 inflammatory axis in spinal cord and DRG, LA coordinately suppresses the expression of TNF-α, HSP-90α, and MMP-13, thereby blocking the neuroinflammation-injury cascade. The finding that LA modulates this pathway adds a new dimension to the understanding of nab-PTX-induced peripheral neuropathy as not just a neuronal but also an immune-mediated pathology. In addition to the IL-17 signaling pathway, other pathways may also be implicated in both the pathogenesis of nab-PTX-induced peripheral neuropathy and the therapeutic effects of LA. For instance, the AGE-RAGE pathway is implicated in oxidative stress and inflammation (44, 45). Similarly, the TNF signaling pathway plays a crucial role in regulating immune responses and apoptosis (46, 47). These signaling pathways are not isolated; they form a complex network that collectively contributes to the alleviation of nab-PTX-induced peripheral neuropathy by LA. For instance, TNF-α is involved in these predicted pathways, highlighting the interconnected nature of these processes. Additionally, HSP90AA1 is involved in the IL-17 signaling pathway, fluid shear stress and atherosclerosis, and NOD-like receptor signaling pathway, further emphasizing the interplay between these pathways. The interplay among these pathways suggests that the development of nab-PTX-induced peripheral neuropathy is a multifactorial process involving oxidative stress, inflammation, and immune responses. The pleiotropic modulation of these key pathways by LA underscores its potential as a multi-target therapeutic strategy.

The findings of this study have significant clinical implications for the management of nab-PTX-induced peripheral neuropathy. Given that the doses of nab-PTX and LA utilized in this study were primarily extrapolated from clinically relevant dosing regimens, it is reasonable to directly apply the standard clinical dosages of LA for the prophylaxis of nab-PTX-induced peripheral neuropathy in clinical practice. In addition, LA is widely used in clinical practice and has a good safety profile, so there is no need to be overly concerned about safety issues. However, there are currently no clinical trials on the prevention of nab-PTX-induced peripheral neuropathy by LA. Future research should focus on validating these findings in clinical settings. Well-designed clinical trials are needed to assess the efficacy and safety of LA in treating nab-PTX-induced peripheral neuropathy.

While our study offers compelling evidence for the therapeutic potential of LA in mitigating nab-PTX-induced peripheral neuropathy, several limitations should be acknowledged. On the one hand, network analysis relies on the existing databases and computational models, which may not fully capture the complexity of biological systems. On the other hand, experimental validation was performed in animal models, which may not fully translate to humans. In addition, we only included male rats in this study. We recognize the importance of integrating sex as a biological variable (SABV) in preclinical research, as mandated by the National Institutes of Health (NIH). The generalizability of our findings regarding the protective effects of LA against nab-PTX-induced neuropathy to females may be limited. This aspect warrants careful consideration when interpreting our results. Future studies systematically investigating the efficacy and mechanisms of LA in both male and female animal models are essential to comprehensively understand its therapeutic potential. Moreover, while our *in vivo* findings robustly demonstrate the association between LA treatment and the modulation of the IL-17 pathway in neural tissues, the present study does not include *in vitro* experiments to elucidate the direct cellular mechanisms. Future studies utilizing cell culture models are essential to confirm the direct cellular targets of LA, delineate the precise molecular interactions, and establish causal relationships within the pathway, for instance, through techniques such as Western blotting, qRT-PCR, and immunofluorescence. Furthermore, we have only experimentally validated the IL-17 signaling pathway; other predicted signaling pathways still need to be verified. Future studies should address these limitations by integrating more comprehensive datasets, conducting clinical studies, investigating the effect of gender on the outcome, conducting *in vitro* experiments, and verifying other predicted signaling pathways.

Despite the above limitations, our integrated approach—combining network toxicology, network pharmacology, molecular docking with *in vivo* validation provides strong, physiologically relevant evidence for the protective role of LA. The consistent downregulation of key pathway components (IL-17, TNF-α, HSP-90α, MMP-13) at the protein level in the target tissues offers compelling support for our findings.

## 5 Conclusion

In conclusion, this study provides a comprehensive understanding of the molecular mechanisms by which LA ameliorates nab-PTX-induced peripheral neuropathy. The findings suggest that LA ameliorates nab-PTX-induced peripheral neuropathy in part by inhibiting the IL-17 signaling pathway in the rat model. Future research should focus on elucidating the direct cellular mechanisms of LA and subsequently translating these mechanistic insights into clinical validation through well-designed trials.

## Data Availability

The original contributions presented in the study are included in the article/**Supplementary Material**. Further inquiries can be directed to the corresponding authors.
