# Supplementary material for: Alpha-lipoic acid ameliorates nab-paclitaxel-induced peripheral neuropathy by inhibiting IL-17 signaling pathway
Source: Front Immunol. 2025 Nov 28;16:1674709. doi: 10.3389/fimmu.2025.1674709 (PMC12698376; doi:10.3389/fimmu.2025.1674709)
Supplement: Supplementary file 1 [file DataSheet1.docx]

**Supplementary Materials**

**Figure S1.** **Changes of body weight in rats.**


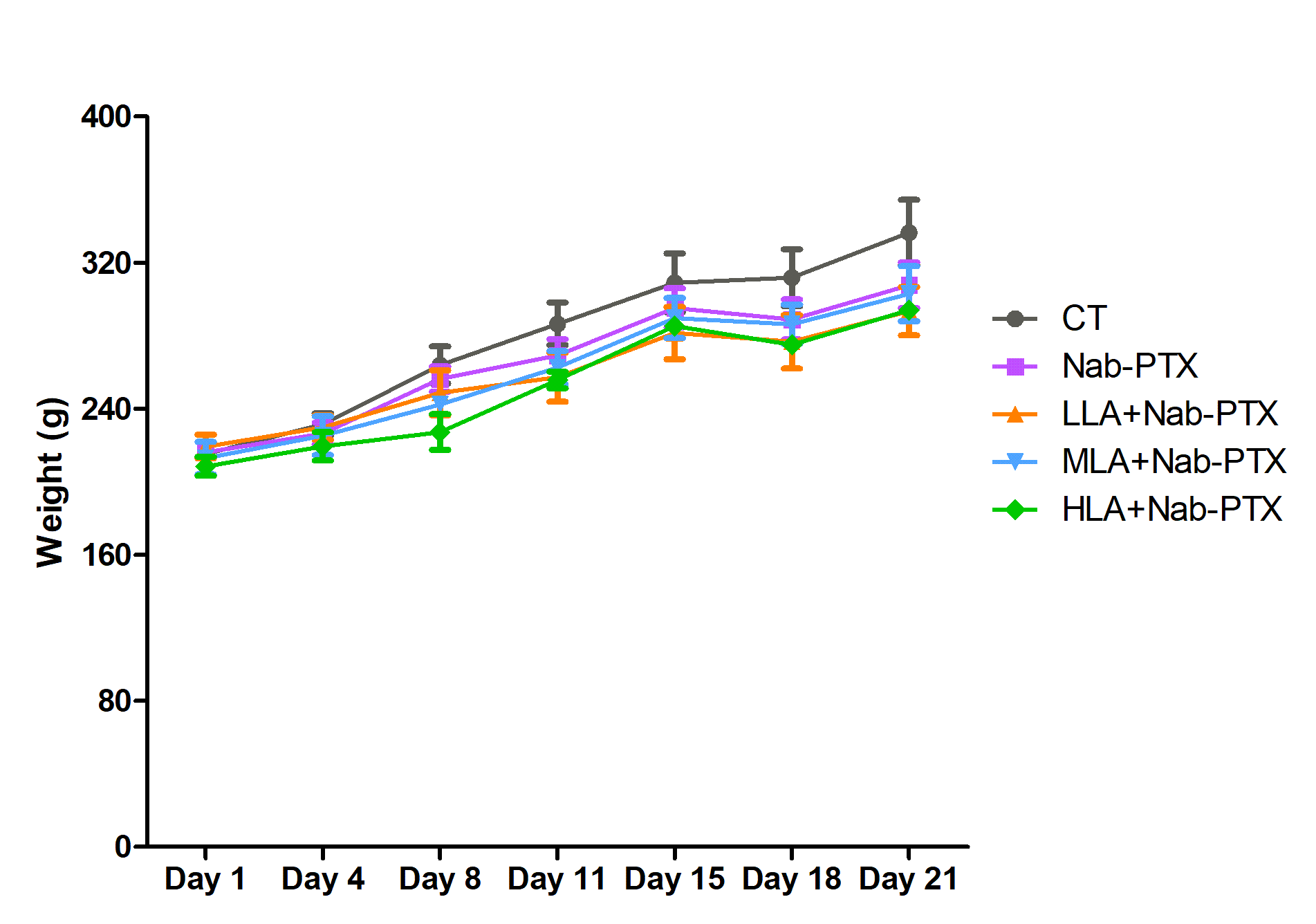


**Table S1. PubChem CID of LA and the PDB IDs of all proteins employed for docking**

| **Drug** | **PubChem CID** |
| --- | --- |
| LA | 864 |
| **Proteins** | **PDB IDs** |
| IL-17 | 4HR9 |
| HSP-90α | 3O0I |
| TNF-α | 6X82 |
| MMP-13 | 2PJT |
